# The relationship of myocardial and liver T2* values with cardiac function and laboratory findings in transfusion-dependent thalassemia major patients: A retrospective cardiac MRI study

**DOI:** 10.34172/jcvtr.2023.31592

**Published:** 2023-06-29

**Authors:** Sepideh Abdi, Negar Taheri, Fatemeh Zahedi Haghighi, Mahya Khaki, Homa Najafi, Mohammad Mehdi Hemmati Komasi, Behrooz Hassani

**Affiliations:** ^1^Cancer Research Center, Cancer Research Institute, Tehran University of Medical Sciences, Tehran, Iran; ^2^Cancer Research Institute, Cancer Institute, Tehran University of Medical Sciences, Tehran, Iran; ^3^Department of Radiology, School of Medicine, Iran University of Medical Sciences, Tehran, Iran; ^4^Faculty of Medicine and Health Sciences, McGill University, Montreal, QC, Canada

**Keywords:** Cardiac MRI, T2* Value, Thalassemia Major, Ventricular Function

## Abstract

**Introduction:**

Cardiac complications are the leading cause of death in thalassemia patients. It is assumed that progressive iron accumulation results in myocyte damage. Myocardial T2* measurement by cardiac MRI quantifies iron overload. We aimed to study the association between left and right ventricular (LV and RV) function and iron deposition estimation by cardiac MRI T2* in a sample of Iranian patients.

**Methods:**

Cardiac MRI exams of 118 transfusion-dependent thalassemia major patients were evaluated retrospectively. Biventricular function and volume and myocardial and liver T2* values were measured. The demographic and lab data were registered. Poisson and chi-square regression analyses investigated the correlation between the T2* value and ventricular dysfunction.

**Results:**

The study participants’ mean (SD) age was 32.7y (9.02), and 47.46% were female. Forty-nine cases (41.52%) revealed at least uni-ventricular dysfunction. LV dysfunction was noted in 20 cases, whereas 47 patients revealed RV dysfunction. The risk of LV dysfunction was 5.3-fold higher in patients with cardiac T2* value less than 10msec (RR=5.3, 95% CI=1.6, 17.1, *P*<0.05). No association was found between age, liver T2* value, serum ferritin level, and chelation therapy with the risk of LV and RV dysfunction.

**Conclusion:**

Cardiac MRI T2* measure is a good indicator of LV dysfunction. Moreover, MRI parameters, especially RV functional measures, may have a substantial role in patient management. Therefore, cardiac MRI should be included in beta-thalassemia patients’ management strategies.

## Introduction

Beta thalassemia is the most common genetic disorder in human populations. ^1^ It is an autosomal recessive disease characterized by the deficiency or lack of normal β-globin chains synthesis.^2,3^ One in 100,000 beta-thalassemia patients is symptomatic. Its prevalence is 23 patients per 100,000 in the Iranian population who have a high carrier frequency of β thalassemia gene.^4,5^

Beta-thalassemia could be classified based on the need for regular blood transfusion. Non–transfusion-dependent thalassemia (NTDT) and transfusion-dependent thalassemia (TDT) are two types of Beta-thalassemia.^6^ Transfusion sustains normal growth in TDT patients.^7,8^

Progressive iron accumulation occurs in different organs in TDT patients. Iron is toxic for many tissues and could lead to cardiac disorders, liver diseases, growth retardation, and multiple endocrine abnormalities. Cardiac events are still the principal cause of mortality and morbidity in beta-thalassemia patients.^3,4,8,9^ Therefore, the timely onset of iron chelation therapy is required to control the rate of iron accumulation. Iron chelation therapy could decrease the non-transferrin bound iron (NTBI) as an oxidative agent that has toxic effects on tissues.^10^ Proper iron overload monitoring is an essential part of patient management. Liver biopsy is known as the most accurate tool to assess iron overload. However, it is invasive and unable to provide a reliable and precise measurement of cardiac iron overload.

Cardiac magnetic resonance (CMR) imaging is the gold standard to calculate ventricular ejection fraction (EF), mass, and volumes.^11^ It is a powerful non-invasive method to measure iron deposition in organs (e.g., heart, liver, and pancreas) and demonstrates cardiotoxicity and myocardial injuries. Moreover, it could help physicians design more efficient therapies for patients to improve the prognosis.^12-19^ As in some other cardiac diseases, MRI of the heart in thalassemia may eliminate the need for a biopsy.^20^

Early detection of cardiac iron accumulation is essential to avoid irreversible cardiac damages such as restrictive cardiomyopathy.^21^ If initiated early, some cardiac complications are often reversible with aggressive intravenous iron chelation therapy. One of the most effective methods to detect cardiac iron levels is T2* CMR imaging. It is a non-invasive method that dose does not require gadolinium contrast injection. Long-term follow-up imaging with T2* has improved the prognosis in thalassemia patients.^22-25^

 In the current study, emphasizing the importance of cardiac dysfunction in TDT patients, we mainly aimed to investigate the association between left and right ventricular (LV and RV) function measured by cardiac MRI and iron deposition estimation by CMR T2* in a sample of Iranian TDT patients.

## Materials and Methods

###  Study design and participants

 We performed a retrospective cross-sectional study on data from 159 thalassemia major patients who were referred to the Rajaei Cardiovascular Medical and Research Center for cardiac MRI from June 2014 to October 2019. Our investigation included one hundred and eighteen patients with long-term transfusion as their treatment. All NTDT cases and patients with pre-existing disorders (diabetes mellitus, lung diseases, and endocrine abnormalities) were excluded. Moreover, we excluded examinations with significant valvular abnormality, poor image quality, significant arrhythmia, presence of the cardiac device, and ischemic cardiomyopathy. The ethics committee waived the need for informed consent due to the study’s retrospective design.

###  Outcome and exposure definitions 

We considered EF < 50% as ventricular dysfunction. This cutoff was selected regarding available guidelines and the fact that thalassemia is a high cardiac output condition.^26^

Mean serum ferritin levels were divided into three groups as follows: acceptable value (lower than 1000ng/ml), mild-moderate increase in ferritin (between 1000 and 2500 ng/mL), and severely increased serum ferritin level (values over > 2500 ng/mL).^27^

We considered liver T2* value below 11.4 milliseconds (msec) as liver iron overload with values of 11.4-3.8 msec: mild, 3.8-1.8 msec: moderate, and < 1.8 msec: severe.^28^

The CMR T2* values lower than 10msec were defined as severe myocardial iron accumulation, while amounts between 10-20msec and measures over 20msec were classified as mild to moderate and no myocardial iron overload, respectively.^29^

###  MRI protocol

 The images were acquired using a 1.5-Tesla MRI scanner (Siemens Avanto, Erlangen, Germany) with an 8-element cardiac-phased array receiver surface coil.

 ECG-gated cine images were obtained during end-expiratory breath-hold. The cardiac size and systolic function were assessed utilizing a set of vertical and horizontal long-axis slices and a stack of cine short-axis images from the ventricular inflow to the apex (imaging matrix: 156 * 192, FOV: 300 mm, slice thickness = 8 mm, without interslice gaps for short-axis images, repetition time/ echo time = 31/1.2 msec). RV and LV volume and ejection fraction (EF) were calculated by determining end-diastolic and end-systolic endocardial contours on cine short-axis images.

**Figure 1:**
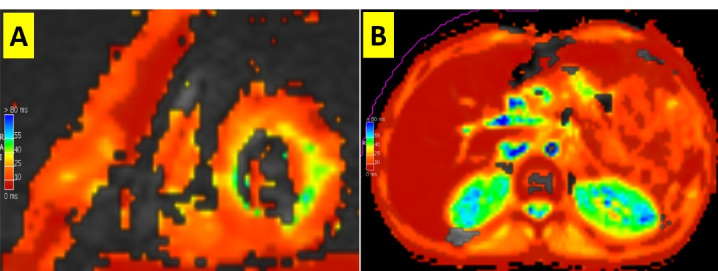
Myocardial (A) and liver (B) MRI T2* mapping images reveal mild to moderate myocardial and severe liver iron overload. Note the map guide on the right side of each image

Myocardial T2* images were acquired in a single breath-hold at the mid-ventricular level in a short-axis view applying a gradient-echo sequence during different echo times (TR = 120 msec). Cardiac T2* image analysis was done in a single mid-ventricular short-axis slice by selecting a large region of interest in the interventricular septum, excluding zones in contiguity to the coronary veins (Figure 1A).

Multi-slice trans-axial sequences (20-echo) of the liver were acquired to determine the liver T2* value (Figure 1B).

###  Statistical analysis 

 Continuous variables were described utilizing mean and standard deviation (SD), while the categorical variables were explained as frequencies and percentages.

The Poisson regression analysis was applied to investigate the association between ventricular dysfunction and the amount of T2*. We first assessed all associations using simple Poisson and Chi-square regression for model fitting. The variables with *P* value < 0.1 were entered into the multiple regression model. We reported the incidence relative risk (IRR) and associated 95% CI for all the investigated variables. All statistical analysis was performed using Stata software (Ver14.1, College Station, Texas, USA). *P *values < 0.05 were considered significant.

## Results

The study was conducted on 118 TDT patients with thalassemia major. The study participants’ mean (SD) age was 32.7y (9.02), and 47.46% were female. All demographic, clinical, and MRI data were abstracted in Table 1.

**Table 1:** Summary of demographic, clinical and MRI data in study participants

**Variable**	**Value**
Female gender, n (%)	56 (47.46%)
Age, Mean (SD)	32.7 (9.02)
Serum ferritin (ng/L), mean (SD)	2437.4 (3762.8)
Under 1000 (ng/L), n (%)	45(41.6%)
1000-2500 (ng/L), n (%)	33(30.5%)
2500 and over (ng/L), n (%)	30(27.7%)
MCV, Mean (SD)	80.7 (6.2)
Myocardial T2^*^ (ms), mean (SD)	23.2 (11.1)
20 ms and over, n (%)	79(66.9%)
10-20 ms, n (%)	16(13.5%)
Under 10 ms, n (%)	23(19.4%)
Liver T2^*^ (ms), mean (SD)	7.0 (7.3)
Over 11.4ms/normal, n (%)	30(25.4%)
3.8-11.4ms/mild, n (%)	25(21.19%)
1.8-3.8ms/moderate, n (%)	24(20.3%)
< 1.8ms/severe, n (%)	39(33.0%)
RVEF in MRI, mean (SD)	51.0 (22.2%)
LVEF in echocardiography, mean (SD)	53.1 (7.1%)
LVEF in MRI, mean (SD)	54.3(9.3%)
LV dysfunction, n (%)	20(16.9%)
RV dysfunction, n (%)	47(39.8%)
Treatment, n (%)	
No drug, n (%)	26 (22%)
One drug, n (%)	52 (44.1%)
Deferasirox, n (%)	16(30.8%)
Deferiprone, n (%)	22(42.3%)
Desferal, n (%)	14(26.9%)
two-drug regime, n (%)	39 (33.1%)
Three-drug regime, n (%)	1 (0.85%)
Spleen removal, n (%)	64(54.2%)
Total, n (%)	118 (100%)

n: number, SD: standard deviation, MCV: mean corpuscular volume, ms: milliseconds, RV: right ventricle, EF: ejection fraction, MRI: magnetic resonance imaging, LV: left ventricle

The mean ± SD of LVEF in T2* categories were 56.27 ± 6.52 for T2* > 20ms, 53 ± 4.26 for T2* between 10-20ms, and 49.27 ± 15.07 for T2* < 10ms (*P* value = 0.004). The mean ± SD of RVEF in T2* categories were 52.6 ± 9 for T2* > 20ms, 49.68 ± 6.79 for T2* between 10-20ms, and 47.54 ± 15.95 for T2* < 10ms (*P *value = 0.1). Results of the Post Hoc test revealed a significant change in LVEF with the decrease of T2* to less than 20 ms.

Overall, 22% of our patients received no chelation therapy during their course of treatment, whereas 44.1% underwent one drug, 33.1% received two chelators, and 0.85% (one patient) underwent a triple-drug regime. Spleen removal had taken place in about 54.2% of the patients (Table 1).

In general, 49 cases (41.52%) with at least uni-ventricular dysfunction were observed (15 men and five women with LV systolic dysfunction, 32 men and 15 women with RV dysfunction). According to multiple Poisson regression, the risk of LV dysfunction was 5.3-fold higher in patients with cardiac T2* value less than 10msec. The observed association was statistically significant (RR = 5.3, 95% CI = 1.6, 17.1). Although the incidence of RV dysfunction was higher in patients with cardiac T2* less than 10ms, it did not show a significant statistical relationship. RV dysfunction showed significant statistical relationship with male gender (RR = 0.5, 95% CI = 0.2, 1.0) but there was no association between LV dysfunction and gender. No association was found between age, liver T2* value, serum ferritin level, and chelation therapy with the risk of LV and RV dysfunction (Table 2).

**Table 2:** Results of the Poisson regression for cardiac dysfunction

**Variable**	**n (%) with LV dysfunction**	**IRR (95% CI)**	***P*** **-value**	**n (%) with RV dysfunction**	**IRR (95%CI)**	***P*** ** value**
Gender						
Male	15 (12.71%)			32 (27.11%)		
Female	5 (4.23%)	0.4 (0.1, 1.3)	0.1	15 (12.71%)	0.5 (0.2, 1.0)	0.05
Age group						
< 20 years	2	Reference		5	Reference	
20-30 years	6	0.9 (0.1, 5.1)	0.9	10	0.6 (0.2, 1.9)	0.4
30-39 years	9	1.4 (0.2, 7.2)	0.6	25	0.8 (0.3, 2.5)	0.8
> 40 years	3	2.7 (0.3, 21.0)	0.3	7	0.8 (0.2,2.9)	0.8
Serum ferritin						
< 1000	5	Reference		18	Reference	
1000-2500	9	1.7 (0.4, 6.9)	0.4	11	1.0 (0.4, 2.5)	0.8
> 2500	5	1.2 (0.2, 6.3)	0.7	14	1.6 (0.6, 4.2)	0.6
Myocardial T2^*^						
> 20ms	7	Reference		27	Reference	
10-20ms	2	1.0 (0.2,5.0)	0.9	9	1.2 (0.5, 2.8)	0.6
< 10ms	11	5.3 (1.6,17.1)	**0.005**	11	1.8 (0.8, 4.1)	0.1
Liver T2^*^						
< 1.8	7	Reference		14	Reference	
1.8-3.8	8	1.9 (0.6,5.6)	0.2	12	1.2 (0.5,2.8)	0.5
3.8-11.4	3	1.1 (0.2,5.0)	0.8	7	0.9 (0.3,2.3)	0.8
> 11.4	2	0.6 (0.1, 3.5)	0.592	14	1.4 (0.6, 3.4)	0.3

n: number, LV: left ventricle, IRR: incidence relative risk, RV: right ventricle, *P* < 0.05 statistically significant

Increased serum ferritin level has significant relationship with severely decreased liver and heart T2^*^ value (Table 3).

**Table 3:** Results of chi-square regression for serum ferritin level

**Variable**	**n (%) with fer under1000**	**n (%) with fer 1000-2500**	**n (%) with fer over2500**	***P*** ** value**
Myocardial T2*				
> 20ms	37 (52.1%)	18 (25.3%)	16 (22.5%)	> 0.05
10-20ms	6 (40.0%)	5 (33.3%)	4 (26.6%)	> 0.05
< 10ms	2 (9.09%)	10 (45.4%)	10 (45.4%)	0.012
Liver T2*				
> 11.4	22 (48.89%)	4 (12.12%)	0	> 0.05
3.8-11.4	13 (28.89%)	6 (18.18%)	4 (17.39%)	> 0.05
1.8-3.8	7 (15.56%)	9 (27.27%)	6 (20.0%)	> 0.05
< 1.8	3 (6.67%)	14 (42.42%)	20 (66.67%)	0.000

n: number, fer: ferritin, *P* < 0.05 statistically significant

## Discussion

T2*MRI is a non-invasive and robust indicator for iron deposition in organs like the heart, liver, and pancreas. It is also a reliable tool for physicians to design more efficient therapies to improve disease prognosis and patient survival.^30,31^ CMR also is an accurate tool for ventricular EF calculation. The present study investigated the laboratory and CMR determinants of LV and RV dysfunction in TDT patients.

 The findings are as follows:

1. We found a strong association between lower cardiac T2* and LV dysfunction in beta-thalassemia patients. However, we did not find any association between cardiac T2* and RV dysfunction.
2. There was no association between liver T2* and cardiac dysfunction.
3. Serum ferritin levels can predict the iron overload in the liver and the heart. We observed an association between heart and liver T2* values with serum ferritin levels.

In our investigation, the lower values of cardiac T2* measured with CMR were associated with an increased risk of LV dysfunction. Many other studies have reported the same association.^32-35^ According to previously published studies, utilizing CMR T2* measurement with early cardiac iron overload management can lead to a 45% reduction in death.^34^ It can prevent severe heart dysfunction by using adequate and aggressive chelation therapy at the proper time.

TDT patients will develop progressive iron accumulation that is toxic to many tissues. The physiologic amount of iron in circulation is taken up by transferrin receptors, and an excessive amount of iron becomes NTBI as an oxidative product. In the heart, NTBI binds with ferritin. This complex accumulates in lysosomes for a long time. Before any symptoms appear, cardiac T2* measure can recognize abnormal signals of excessive iron accumulation in the heart. This excessive iron can ruin lysosomal, sarcoplasmic, and mitochondrial membranes and cause ion channel disruption.^36^ It can also cause reversible myocyte failure, dilated cardiomyopathy, decreased LV EF, and arrhythmias.^37,38^

We observed a higher prevalence of RV systolic dysfunction than LV dysfunction in the present investigation, but there was no association between myocardial iron overload measured by CMR T2* with RV systolic dysfunction. It shows that other causes of cardiac problems in thalassemia patients should value more. On the other hand, according to some previous studies, RV dysfunction is the most significant predictor of pulmonary hypertension in thalassemia patients, one of the leading causes of mortality.^39^

We should consider that iron overload is not the only cause of cardiac disease in beta-thalassemia patients. Nutritional deficiencies, genetic variations, pulmonary hypertension, changes in cardiac function due to fibrosis, and a decrease in vascular compliance are other causes that should be mentioned in the heart disease pathway in beta-thalassemia patients. However, they are not as prominent as iron accumulation.^8,32,40^

We found no association between liver T2* value and biventricular dysfunction. These findings are consistent with the reported results of previous studies.^35^ It might be due to proficient chelation therapy and the fact that iron removes more rapidly from the liver than from the heart if proficient chelation therapy is administered.^28,33^ We suppose that liver iron overload is not a reliable determinant of cardiac involvement. This finding underscores the value of CMR T2* measurement in revealing the cardiotoxicity of excessive iron.

A relationship between severe liver and cardiac iron overload and serum ferritin level was detected. It may be due to the mechanism of iron deposition in hepatocytes. When excessive iron saturates plasma transferrin, NBTI appears and is rapidly taken by parenchymal cells of the liver and can cause liver damage and fibrosis.^8^

Although the incidence of LV and RV dysfunction was slightly higher in males than females, we only found a statistically significant association between gender and RV dysfunction. Kirk et al. have also shown that overall cardiac diseases were higher in male patients. However, they observed no significant association in this regard.^33^ The higher incidence of cardiac dysfunction might be due to the daily life activities of men and women,^41^ higher prevalence of heart failure with preserved EF in women than men, and differences in their cardiovascular system. Women compared to men have a smaller heart with lower heart muscle tone, but their LV contractility and mass are preserved better with aging.^42^

Although our results are valuable, we encountered some limitations in our investigation. First, the study’s retrospective design precluded a thorough evaluation of all clinical, lab data, and imaging parameters. Another limitation of our study is that follow-up data are not available. It was a study about the role of T2* imaging and its relationship with cardiac MRI parameters. Further investigations about patient outcomes are worthful. A few previous studies investigated the predictive value of CMR parameters in thalassemia patients,^43^ which is lacking in our research. Furthermore, we have to emphasize that advanced imaging techniques, including cardiac strains, can be valuable in the assessment of patients with thalassemia and should be considered in future studies. Finally, further multicentric studies with a comprehensive MRI protocol including novel mapping techniques and feature tracking measurements may provide valuable information for more efficient management in this patient population.

## Conclusion

 Cardiac MRI has an undeniable role in the management of thalassemia patients. CMR T2* measure is a good indicator of LV dysfunction. Moreover, cardiac MRI can reveal RV dysfunction which has both diagnostic and prognostic values in this group of patients. Therefore, we believe that cardiac MRI should be included in beta-thalassemia patients’ management programs to predict future cardiac complications.

## Acknowledgements

 Not applicable

## Competing Interests

 There is no conflict of interests.

## Ethical Approval

 The ethics committee of Rajaie Cardiovascular Medical and Research Center approved the study. Ethical approval code: IR.RHC.REC.1399.022.

## Funding

 No support was achieved for this study.
